# Whole Blood Transcriptome Profiling Identifies DNA Replication and Cell Cycle Regulation as Early Marker of Response to Anti-PD-1 in Patients with Urothelial Cancer

**DOI:** 10.3390/cancers13184660

**Published:** 2021-09-17

**Authors:** Sandra van Wilpe, Victoria Wosika, Laura Ciarloni, Sahar Hosseinian Ehrensberger, Rachel Jeitziner, Paolo Angelino, Tjitske Duiveman-de Boer, Rutger H. T. Koornstra, I. Jolanda M. de Vries, Winald R. Gerritsen, Jack Schalken, Niven Mehra

**Affiliations:** 1 Department of Medical Oncology, The Radboud University Medical Center, 6500 HB Nijmegen, The Netherlands; Sandra.vanWilpe@radboudumc.nl (S.v.W.); Winald.Gerritsen@radboudumc.nl (W.R.G.); 2 Novigenix SA, 1066 Epalinges, Switzerland; Victoria.Wosika@novigenix.com (V.W.); Laura.Ciarloni@novigenix.com (L.C.); Sahar.Hosseinian@novigenix.com (S.H.E.); 3 Bioinformatics Core Facility, Swiss Institute of Bioinformatics, 1015 Lausanne, Switzerland; Rachel.Jeitziner@sib.swiss (R.J.); Paolo.Angelino@unil.ch (P.A.); 4 Department of Tumor Immunology, Radboud Institute for Molecular Life Sciences, Radboud University Medical Center, 6525 GA Nijmegen, The Netherlands; Tjitske.Duiveman-deBoer@radboudumc.nl (T.D.-d.B.); Jolanda.deVries@radboudumc.nl (I.J.M.d.V.); 5 Department of Medical Oncology, Rijnstate Hospital, 6815 AD Arnhem, The Netherlands; RKoornstra@Rijnstate.nl; 6 Department of Urology, Radboud University Medical Center, 6500 HB Nijmegen, The Netherlands; Jack.Schalken@radboudumc.nl

**Keywords:** urothelial cancer, anti-PD-1, biomarkers, proliferation, transcriptome

## Abstract

**Simple Summary:**

Unfortunately, not all patients with urothelial cancer benefit from checkpoint inhibitors (ICIs). Currently, the first radiological response evaluation is not performed until after 9 to 12 weeks of ICI therapy. Early response biomarkers might enable an early switch to more effective therapies in patients that do not respond. In this study, we aimed to identify early response biomarkers in the blood of patients treated with ICIs. In whole blood of patients with clinical benefit, genes involved in DNA replication and cell cycle regulation were upregulated after 2 to 6 weeks of treatment. This appeared to be a result of T cell proliferation and was not observed in patients without clinical benefit. Our results suggest that whole blood RNA sequencing can contribute to early response prediction in patients treated with ICIs and warrants further research.

**Abstract:**

Although immune checkpoint inhibitors improve median overall survival in patients with metastatic urothelial cancer (mUC), only a minority of patients benefit from it. Early blood-based response biomarkers may provide a reliable way to assess response weeks before imaging is available, enabling an early switch to other therapies. We conducted an exploratory study aimed at the identification of early markers of response to anti-PD-1 in patients with mUC. Whole blood RNA sequencing and phenotyping of peripheral blood mononuclear cells were performed on samples of 26 patients obtained before and after 2 to 6 weeks of anti-PD-1. Between baseline and on-treatment samples of patients with clinical benefit, 51 differentially expressed genes (DEGs) were identified, of which 37 were upregulated during treatment. Among the upregulated genes was *PDCD1*, the gene encoding PD-1. STRING network analysis revealed a cluster of five interconnected DEGs which were all involved in DNA replication or cell cycle regulation. We hypothesized that the upregulation of DNA replication/cell cycle genes is a result of T cell proliferation and we were able to detect an increase in Ki-67^+^ CD8^+^ T cells in patients with clinical benefit (median increase: 1.65%, range −0.63 to 7.06%, *p* = 0.012). In patients without clinical benefit, no DEGs were identified and no increase in Ki-67^+^ CD8^+^ T cells was observed. In conclusion, whole blood transcriptome profiling identified early changes in DNA replication and cell cycle regulation genes as markers of clinical benefit to anti-PD-1 in patients with urothelial cancer. Although promising, our findings require further validation before implementation in the clinic.

## 1. Introduction

Immune checkpoint inhibitors (ICIs) have become an integral part of therapy for patients with metastatic urothelial cancer (mUC). Since a few years, ICIs targeting the programmed cell death protein 1 (PD-1)/ programmed cell death ligand 1 (PD-L1) axis are used to treat cisplatin-ineligible patients with a PD-L1 positive tumor as well as patients that have progressed on first-line platinum-based chemotherapy. Additionally, maintenance therapy with PD-L1 inhibitor avelumab was recently approved for the treatment of patients who achieved a response or stable disease with first-line chemotherapy [1]. Although anti-PD-(L)1 prolongs median overall survival (OS), only a minority of patients benefit from it [1,2]. In a phase III clinical trial, second-line treatment with PD-1 inhibitor pembrolizumab induced responses in 21.1% and disease control in 38.5% of mUC patients [2].

Biomarkers that can predict the clinical outcome to anti-PD-(L)1 are urgently needed. Application of biomarkers limits the use of PD-(L)1 inhibitors in patients that do not benefit from it, thereby preventing immune-related toxicity and enabling the rapid introduction of other, potentially more effective therapies. Several promising treatment strategies have emerged and are either in late-stage clinical trials or already approved by the Food and Drug Administration for the treatment of mUC [3,4,5,6]. Recently approved drugs include enfortumab vedotin [3,4] and erdafitinib [5]. Additionally, dual checkpoint inhibition is currently being studied in various disease settings and might be beneficial in some patients that do not benefit from anti-PD-(L)1 monotherapy [6,7]. 

So far, efforts have focused on the identification of predictive biomarkers that can be obtained prior to treatment initiation. Although tumor mutational burden, PD-L1 expression and CD8^+^ T cell infiltration at baseline appear to enrich for response to ICIs [8,9,10,11,12], these biomarkers are not accurate enough to be used as stand-alone biomarkers. Early response biomarkers may also have clinical utility but have been underexplored. In current practice, the first radiological response evaluation is usually not performed until after 12 weeks of ICI therapy and is sometimes equivocal. Clinically stable patients with suspected progression may continue treatment after the first scan according to iRECIST to avert treatment discontinuation in patients with delayed responses or pseudo-progression [13,14,15]. Early blood-based response biomarkers may provide a reliable way to determine whether ICIs are effective before imaging is available and can be particularly useful for those with equivocal imaging.

Translational studies in patients with various tumor types indicate that clinical benefit to ICIs is accompanied by systemic immunological changes during the first weeks of treatment. In patients with melanoma or lung cancer, decreases in IL-6 and IL-8 during the first weeks of therapy have been associated with an improved outcome to ICIs [16,17]. Additionally, a study in patients with melanoma or Merkle cell carcinoma demonstrated that a high frequency of circulating PD-1^+^ TIGIT^+^ CD8^+^ T cells after 1 month of anti-PD-1 was associated with an increased response rate and longer OS [18]. Furthermore, studies in lung cancer and melanoma have described an association between T cell proliferation and response to therapy [19,20]. However, data on ICI-induced changes in peripheral blood of mUC patients are lacking.

Although flow cytometry and single-cell RNA sequencing may provide insights into the biological mechanisms underlying the response to ICIs, these analyses are laborious and/or costly, limiting its potential for early response evaluation in the clinic. Interestingly, in melanoma patients treated with cytotoxic T-lymphocyte-associated protein 4 (CTLA-4) inhibitor tremelimumab, an association was observed between immune-related toxicity and whole blood RNA expression after 30 days of treatment [21]. This raises the question of whether early changes in whole blood RNA transcripts can also be used for the identification of responders, a test that would be relatively easy to implement in the clinic.

We conducted an exploratory study aimed at the identification of early markers of response to anti-PD-1 in patients with mUC. By performing a comprehensive, unbiased whole blood transcriptome analysis, we reveal that DNA replication/cell cycle genes and *PDCD1*, the gene encoding for PD-1, are upregulated in patients with clinical benefit to ICIs but not in patients who progress within 6 months. We show that the upregulation of DNA replication/cell cycle genes is paralleled by an increase in Ki-67^+^ CD8^+^ T cells, suggesting that this upregulation is partly due to the proliferation of CD8^+^ T cells. 

## 2. Materials and Methods

### 2.1. Patients

This retrospective study included 32 patients with mUC who were treated with anti-PD-1 in the Radboud University Medical Center between 2017 and 2019. Patients were treated with nivolumab 3 mg/kg every 2 weeks or pembrolizumab 200 mg every 3 weeks. During treatment, patients were evaluated according to RECIST1.1 [22]. Patients were considered to have clinical benefit if they had a radiological and clinical progression-free survival (PFS) of at least 6 months.

All patients provided informed consent for the use of biomaterials as approved by the medical ethics committee of the Radboud University Medical Center (project number NL60249.091.16). This study was performed in accordance with relevant guidelines and regulations.

### 2.2. Blood Collection and Processing

Blood was drawn prior to the first three cycles of anti-PD-1 therapy (i.e., at 0, 2 and 4 weeks for nivolumab and at 0, 3 and 6 weeks for pembrolizumab). At these timepoints, a complete blood cell count was performed as part of routine clinical care. In addition, blood was collected in one PAXgene Blood RNA tube (BD Biosciences, San Jose, CA, USA) and three 10 mL EDTA tubes. PAXgene tubes were stored at −80 °C until RNA purification. Peripheral blood mononuclear cells (PBMCs) were isolated using Ficoll gradient centrifugation. After adding Ficoll (Lymphoprep^TM^, Axis-Shield, Dundee, UK), samples were centrifuged at 750 g for 20 min at room temperature without brake. The PBMC layer was transferred to a new tube and washed with phosphate-buffered saline (PBS). Viable cells were counted using a LUNA FL dual fluorescence cell-counter (Logos Biosystems). Cells were resuspended in freezing medium (10% DMSO, 90% fetal bovine serum), at a concentration of 5 × 10^6^ viable cells per ml and stored in liquid nitrogen. A baseline sample and the earliest on-treatment sample available were used for subsequent analyses.

### 2.3. Whole Blood RNA Sequencing

Total RNA was extracted from whole blood using the PAXgene blood miRNA kit (Qiagen, Venlo, Netherlands). RNA quantity was determined using Qubit (Thermo Fisher Scientific, Waltham, MA, USA). RNA quality was assessed on a Bioanalyzer (Agilent Technologies, Santa Clara, CA, USA). Samples with a RIN below six were excluded from the analysis. Per sample, at least 500 ng of total RNA was used for library preparation.

RNA samples were treated for globin and ribosomal RNA depletion with the Illumina Globin-Zero Gold kit (Illumina, San Diego, CA, USA). Library preparation was performed with the Illumina TruSeq RNA Library Prep Kit v2. Sequencing was performed on Illumina NovaSeq 6,000 (non-stranded, paired-end 2 × 150 bp) with an estimated average output of 20–30 million reads/sample.

Sequence adapters (Illumina TruSeq) were trimmed using Atropos (1.1.21), with the following base quality threshold and adapters: —quality-base 33 -a ′AACACTCTTTCCCT′ -a ′AGATCGGAAGAGCG′ -a ′AGGGAAAGAGTGTT′ -a ′CGCTCTTCCGATCT′ —overlap 8. The trimmed paired-end reads were used as input for gene expression analysis on the LITOSeek^®^ platform (Novigenix SA, Epalinges, Switzerland). Reads were aligned to the human reference hg38 with HISAT2 (version 2.1.0), and the Salmon algorithm (version 0.13.1) was used to quantify transcript expression. A preliminary quality check was performed using the MultiQC tool (version 1.8).

The quantified transcript expression data was used to identify early markers of response. Differential expression analyses (DEA) were performed using DESeq2 to identify differentially expressed genes between paired baseline and on-treatment samples in patients with and without clinical benefit. A multi-factor design was used to account for differences between patients while estimating the effect of anti-PD-1 therapy. Log fold changes and adjusted *p*-values (padj) were determined for all genes using a Wald test with Benjamini–Hochberg correction. Functional enrichment analysis was performed with EnrichR, using the Reactome 2016 database [23,24]. Network analyses were performed using STRING [25]. In both DEAs and functional analyses, an adjusted *p*-value ≤ 0.05 was considered significant. Correlations between changes in different genes were analyzed with the corrplot package from GitHub (version 0.84) using the Pearson’s correlation coefficient [26]. Kaplan–Meier curves were generated to display differences in PFS between patients with an above-median versus below-median increase in genes of interest.

### 2.4. Flow Cytometry

Flow cytometry was used to analyze the expression of CD3, CD4, CD8, CTLA-4 (CD152), LAG-3 (CD223), PD-L1 (CD274), PD-1 (CD279), TIM-3 (CD366), HLA-DR, and Ki-67 on PBMCs. For antibody details, we refer to Table S1.

PBMCs were thawed rapidly in a 37 °C water bath and diluted in RPMI 1,640 medium. Cell number and viability were determined with a hemocytometer using trypan blue. The cells were then kept with fixable viability dye efluor 780 (eBioscience, San Diego, CA, USA) diluted in PBS for 30 min at 4 °C. Subsequently, the antibodies for cell surface staining were added (all antibodies except anti-Ki-67). These were diluted in brilliant staining buffer (BD Biosciences, San Jose, CA, USA). The cells were incubated with the antibody mix for 30 min at 4 °C in the dark. For the intracellular staining of Ki-67, cells were then fixed with Fix/Perm (eBioscience) for 2 h at 4 °C. After washing, the cells were resuspended in permeabilization buffer containing the Ki-67 antibody and incubated for 30 min at 4 °C.

The staining intensity was measured with the FACSLyric (BD Biosciences). Instrument settings were verified and adjusted before each acquisition using single stainings. Data were analyzed with FlowJo Software (Tree Star Inc., Ashland, OR, USA). Positive and negative cell populations for each marker were determined using fluorescence minus one (FMO) controls. The gating strategy is shown in Figure S1. Markers expressed on <1% of the cell of interest were excluded from the analyses.

A Wilcoxon test was used to compare early changes in Ki-67^+^ CD8^+^ and Ki-67^+^ CD4^+^ cells between baseline and on-treatment samples in patients with and patients without clinical benefit. A *p*-value ≤ 0.05 was considered significant. Correlations between changes in gene expression and changes in Ki-67^+^ CD8^+^ T cells were analyzed using Pearson’s correlation coefficient. Descriptive statistics were used to describe checkpoint molecule and HLA-DR expression on these cell subsets. 

## 3. Results

### 3.1. Patient Cohort

In total, 32 patients with mUC were included. Most patients were treated with pembrolizumab (78.1%) and received anti-PD-1 as second-line treatment (65.6%). Patient characteristics are summarized in Table 1. 

Nineteen patients experienced clinical benefit (59.4%). Five of them had a complete response, 12 a partial response, and one had stable disease according to RECIST1.1 [22]. Additionally, one patient was non-evaluable according to RECIST1.1 criteria but showed a decrease in FDG uptake on PET imaging. The median PFS in the group with clinical benefit was 25 months (range: 10 to > 42). The median OS could not be determined because only two patients had died at the last follow-up (median follow-up: 33 months). By contrast, thirteen patients (40.6%) did not experience clinical benefit. None of these patients had an initial response. In these patients, median PFS and OS were 2 (range: 1 to 3) and 6 months (range: 1 to 30), respectively.

In most patients, the on-treatment sample was collected after one cycle of anti-PD-1 (75%). In total, high-quality RNA-sequencing data of baseline and on-treatment samples was available for 26 of 32 patients (14 with clinical benefit, 12 without clinical benefit). In five patients, either the baseline or on-treatment sample did not pass the quality check. In one patient, no PAXgene tube was available. PBMCs were available of 30 patients (18 with clinical benefit, 12 without clinical benefit).

### 3.2. Whole Blood Transcriptome Changes in Patients with and without Clinical Benefit

**Figure 1:**
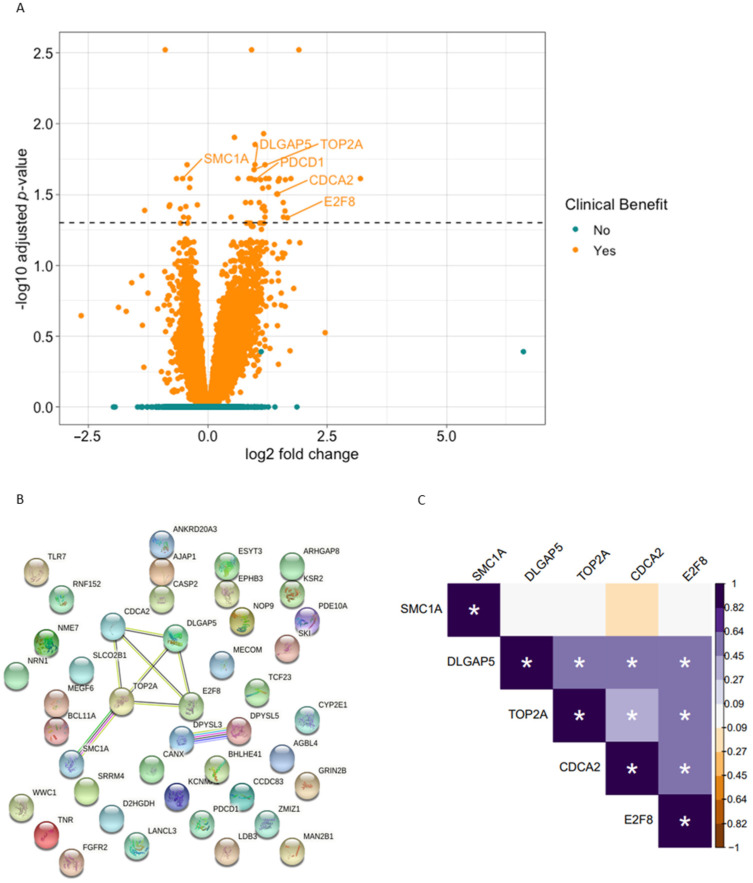
Differentially expressed genes (DEGs) between baseline and on-treatment samples. (**A**) Volcano plot of DEGs in patients with (orange) and without clinical benefit (green). The dashed line indicates the significance threshold (padj = 0.05). In patients with clinical benefit, 51 DEGs were identified. No significant genes were found in patients without clinical benefit. (**B**) STRING protein-protein interaction analysis on the DEGs identified in patients with clinical benefit. Yellow lines indicate text mining evidence, black lines co-expression evidence, green lines neighborhood evidence, blue line co-occurrence evidence, purple line experimental evidence and light blue lines database evidence. (**C**) Correlation heatmap summarizing the correlations between expression levels of the identified cell cycle/proliferation genes. Baseline and on-treatment samples of both patients with and without clinical benefit were included in this analysis. Colors refer to the Pearson’s correlation coefficient. Asterisks indicate a *p*-value ≤ 0.05.

First, a DEA between baseline and on-treatment samples was performed in 14 patients with clinical benefit to anti-PD-1. Fifty-one differentially expressed genes (DEGs) were identified, of which 37 were upregulated and 14 downregulated (Figure 1A). The average fold change of these DEGs was 2.0. For biological interpretation of the identified DEGs, we first generated a protein-protein interaction network using STRING to explore interactions between the identified protein-coding DEGs. Among the 51 DEGs were 43 protein-coding genes. STRING network analysis revealed a cluster of five interconnected DEGs which were all involved in DNA replication or cell cycle regulation (Figure 1B). Four of these genes were upregulated, i.e., *DLGAP5*, *TOP2A*, *CDCA2,* and *E2F8*. Changes in the expression of these genes were highly correlated (Figure 1C and Figure S2). *SMC1A*, on the other hand, which is known for its role in chromosome cohesion during the cell cycle, was downregulated and changes in *SMC1A* poorly correlated with changes in the other DNA replication/cell cycle genes in our cohort. Pathway enrichment analysis did not identify any significantly enriched pathways (padj ≤ 0.05). As no enriched pathways were identified, we looked further into the function of individual DEGs. Interestingly, we observed that *PDCD1,* the gene that encodes PD-1, was upregulated in patients with clinical benefit. Except for *PDCD1*, the identified DEGs did not have an established role in immunology. All identified DEGs together with their log fold changes and *p*-values are listed in the Supplemental file.

Subsequently, we performed a similar analysis in the 12 patients without clinical benefit. In contrast to the patients with clinical benefit, no DEGs were identified in these patients (Figure 1A). Particularly, no net increase or decrease was observed in any of the DNA replication/cell cycle genes that were differentially expressed in the patients with clinical benefit, nor in *PDCD1* expression (Figure S3).

**Figure 2:**
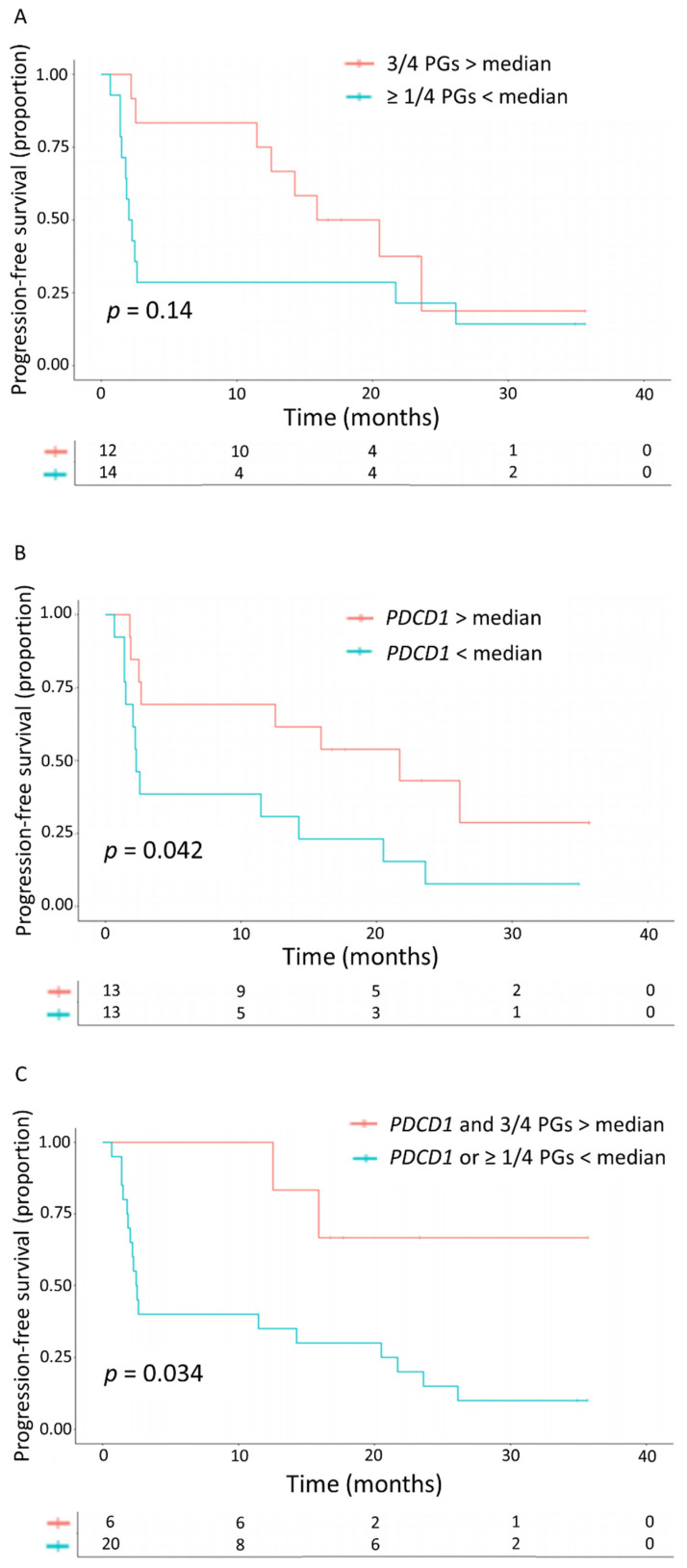
Kaplan-Meier curves. (**A**) Progression-free survival in patients with versus without an above-median increase in three of the four DNA replication/cell cycle genes that were upregulated in the patients with clinical benefit (PGs; *DLGAP5*, *TOP2A*, *CDCA2*, and *E2F8*). (**B**) Progression-free survival in patients with versus without an above-median increase in *PDCD1*. (**C**) Progression-free survival in patients with versus without an above-median increase in three proliferation genes and *PDCD1*.

To illustrate how well these DEGs discriminate between patients with and without clinical benefit, PFS curves were generated. For the DNA replication and cell cycle genes, patients were dichotomized based on whether or not there was an above-median increase in at least three of the four DNA replication/cell cycle genes that were found to be upregulated in patients with clinical benefit. The genes were combined as there was a strong correlation between the four genes. An above-median increase in at least three genes (instead of one, two, or four) was chosen as the cut-off because clinical benefit was observed in approximately half of the cohort and this approach split the cohort into two equally sized groups. Six-month PFS was better in patients with an above-median increase in at least three of the DNA replication/cell cycle genes (83.3% versus 28.6%; *p* = 0.14, Figure 2A). The difference in PFS between patients with and without an above-median increase in *PDCD1* was less pronounced (69.2% versus 38.5%, *p* = 0.042, Figure 2B). All six patients with both an above-median increase of at least three DNA replication/cell cycle genes and *PDCD1* were progression-free at 6 months (Figure 2C).

### 3.3. Cell Specificity of the Identified DEGs

Based on the mechanism of action of anti-PD-1 and previously published data describing T-cell reinvigoration in responders to ICI, we hypothesized that the upregulation of DNA replication genes/cell cycle genes in patients with clinical benefit may be partly due to the proliferation of peripheral T cells. To evaluate the cell specificity of the identified DNA replication/cell cycle genes, we used a publicly available dataset consisting of RNA-sequencing data of flow cytometry-sorted PBMCs (GSE107011 [27]). We observed enhanced expression of *DLGAP5*, *TOP2A*, *CDCA2,* and *E2F8* in T cells compared to unsorted PBMCs. Expression was particularly high in CD8^+^ effector memory cells, T-helper 1 cells, follicular helper T cells, and regulatory T cells. *SMC1A*, on the other hand, was highly expressed in nearly all immune cells subsets (Figure S4), showing no specificity for any particular immune cell subset.

### 3.4. Changes in Lymphocyte Proliferation

**Figure 3:**
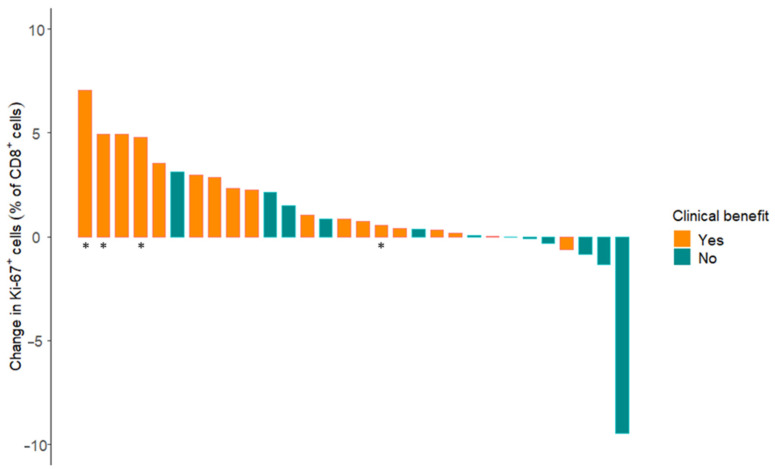
Changes in Ki-67 expression on CD8^+^ T cells during anti-PD-1 therapy. A significant difference was found between patients with (orange) versus patients without clinical benefit (green, *p* = 0.010). Asterisks indicate patients with a complete response.

We then used complete blood cell counts and flow cytometry to study changes in lymphocytes counts and proliferation during anti-PD-1 therapy. No changes were seen in absolute lymphocyte counts nor in the percentage of CD3^+^, CD8^+^ or CD4^+^ T cells in peripheral blood of patients with clinical benefit (Figure S5). Nevertheless, the percentage of CD8^+^ T cells that expressed proliferation marker Ki-67 increased during the first weeks of treatment in patients with clinical benefit (median increase: 1.65%, range −0.63–7.06%, *p* = 0.012). An increase in Ki-67^+^ CD8^+^ T cells was not observed in patients without clinical benefit (*p* = 0.71). When we directly compared the changes between patients with and without clinical benefit, we also found a significant difference (Figure 3, *p* = 0.010). This was still the case when an outlier in the group without clinical benefit (7.06% decrease in Ki-67^+^ CD8^+^ T cells) was removed (*p* = 0.021). No significant change in the percentage of Ki-67^+^ CD4^+^ T cells was observed. As shown in Figure S6, median PFS was numerically longer in the group with an above-median increase in Ki-67^+^ CD8^+^ T cells.

We then correlated the changes in Ki-67^+^ CD8^+^ T cells with the changes in DNA replication and cell cycle genes in the 24 patients for which we had both data available. There was a positive correlation between changes in Ki-67^+^ CD8^+^ T cells and changes in *CDCA2* (R = 0.42, *p* = 0.04) or *DLGAP5* (R = 0.56, *p* = 0.004). A trend for a positive correlation was observed between changes in Ki-67^+^ CD8^+^ T cells and changes in *E2F8* (R = 0.34, *p* = 0.10) and *TOP2A* (R = 0.34, *p* = 0.10), but these correlations were not statistically significant (Figure S7).

### 3.5. Phenotyping of Ki-67^+^ CD8^+^ T Cells

**Figure 4:**
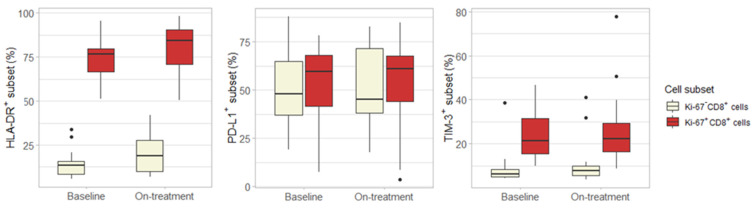
Differences between Ki-67^+^ CD8^+^ T cells and Ki-67^−^ CD8^+^ T cells. Expression of HLA-DR, PD-L1 and TIM-3 is higher on Ki-67^+^ CD8^+^ cells compared to Ki-67^−^ CD8^+^ T cells. Nevertheless, baseline and on-treatment expression does not clearly differ, suggesting that the differences between Ki-67^+^ and Ki-67^−^ CD8^+^ T cells are not treatment-related.

As there are currently no PD-1 antibodies available that can reliably stain PD-1 on T cells after treatment with pembrolizumab or nivolumab, we could not use flow cytometry to confirm an increase in PD-1^+^ T cells in patients with clinical benefit [28]. To further explore the activity of the Ki-67^+^ CD8^+^ T cells in patients with clinical benefit, we assessed the expression of activation markers on Ki-67^+^ CD8^+^ T cells. We observed that the majority of Ki-67^+^ CD8^+^ T cells were positive for HLA-DR in contrast with Ki-67^−^ CD8^+^ T cells, which were mostly negative for HLA-DR. TIM-3 expression was also higher on Ki-67^+^ CD8^+^ T cells, but no clear difference was observed in PD-L1 expression (Figure 4). As CTLA-4 and LAG-3 were expressed on less than 1% of CD4^+^ and CD8^+^ T cells, differences between Ki-67^+^ CD8^+^ T cells and Ki-67^−^ CD8^+^ T cells for these checkpoint molecules could not be assessed. Importantly, the difference in HLA-DR and TIM-3 between Ki-67^+^ CD8^+^ T cells and Ki-67^−^ CD8^+^ T cells did not appear to be treatment-related, as there was no clear difference between baseline expression and on-treatment expression of these markers. 

## 4. Discussion

In this pilot study, we performed a genome-wide analysis of whole blood RNA expression to identify early markers of response to anti-PD-1 in patients with mUC. In patients deriving clinical benefit from anti-PD-1, upregulation of several DNA replication/cell cycle genes and *PDCD1* was observed during the first weeks of ICI therapy. These changes did not occur in patients with initial disease progression. Using flow cytometry, we subsequently demonstrated an early increase in Ki-67^+^ CD8^+^ T cell in peripheral blood of patients with clinical benefit, suggesting that the upregulation of DNA replication/cell cycle genes is partly due to the proliferation of CD8^+^ T cells. 

Whereas our study is the first to evaluate ICI-induced changes in peripheral blood of mUC patients, upregulation of cell cycle genes and increases in Ki-67^+^ CD8^+^ T cells during anti-PD-1 therapy have previously been described in other tumor types. A study in melanoma patients reported upregulation of cell cycle genes in peripheral CD8^+^ T cells after 21 days of ICI therapy [29]. Among these were the four genes that were significantly upregulated in patients with clinical benefit in our study (*DLGAP5*, *TOP2A*, *CDCA2* and *E2F8*). Furthermore, previous studies have shown that an early increase in peripheral Ki-67^+^ CD8^+^ T cells is associated with response to anti-PD-1 in patients with lung cancer or melanoma [19,20]. 

As we analyzed the whole blood transcriptome, we cannot ascertain which cells are responsible for the observed upregulation of DNA replication/cell cycle genes in patients with clinical benefit. We postulated that proliferating T cells are responsible for the increase in DNA replication/cell cycle genes and performed flow cytometry experiments to confirm that T cell proliferation increases in patients with clinical benefit. Although *MKI67* RNA expression was not upregulated in our cohort, i.e., the transcript encoding the Ki-67 protein, we decided to use Ki-67 as a marker for T cell proliferation. Ki-67 is an established proliferation marker and the absence of *MKI67* upregulation might be explained by the fact that Ki-67 protein expression is partly regulated by proteasomal degradation [30]. We were able to detect an increase in Ki-67^+^ CD8^+^ T cells in patients with clinical benefit, supporting the idea that proliferating T cells are accountable for the observed changes in DNA replication/cell cycle genes. Our findings that the expression of *DLGAP5*, *TOP2A*, *CDCA2* and *E2F8* is enhanced in T cells, along with the observed correlation between changes in DNA replication/cell cycle genes and changes in Ki-67^+^ CD8^+^ T cells further endorses this. Naturally, CD8^+^ T cells account for only a small part of the total RNA pool of whole blood. This could explain why there was only a weak correlation between changes in Ki-67^+^ CD8^+^ T cells and changes in *E2F8* or *TOP2A* expression. It could also explain why individual DNA replication/cell cycle genes were upregulated in patients with clinical benefit but no significant enrichment for DNA replication or cell cycle pathways was found. Taken together, it is promising that we were able to detect signs of proliferation in the blood of patients with clinical benefit as early as 2 to 6 weeks into treatment.

Besides the changes in proliferation markers, we also observed a significant increase in *PDCD1* RNA expression in patients with clinical benefit. This finding is in line with previous studies showing that anti-PD-1 primarily induces proliferation of progenitor exhausted T cells, i.e., cells that express PD-1 [31,32]. Unfortunately, we could not use flow cytometry to confirm an increase in PD-1^+^ T cells in patients with clinical benefit due to competitive binding/steric hindrance of the flow cytometry antibody with pembrolizumab and nivolumab binding sites [28]. Nevertheless, the selective upregulation of *PDCD1* expression in patients with clinical benefit suggests that this gene might function as an early biomarker of response to anti-PD-1, together with the identified DNA proliferation/cell cycle genes. 

To the best of our knowledge, this is the first study to evaluate early response biomarkers for ICI therapy in mUC. We were able to detect signs of proliferation in the peripheral blood of patients with clinical benefit using two different assays, focused on different biological levels, i.e., RNA and protein expression. Nevertheless, this study has some limitations. Firstly, we decided to analyze whole blood transcripts because this is much easier to implement in the clinic compared to RNA sequencing of isolated cell subsets. The latter, however, might have provided more insight into the activity of individual peripheral blood cell subsets. Secondly, our study population was small. Further research is needed to validate our results and to determine whether whole blood expression of a selected set of genes can be used in the clinic as an early response biomarker in mUC. 

Our findings indicate that early changes in proliferation genes and PDCD1 might function as early response biomarkers in mUC. Early response biomarkers enable an early switch to other, potentially more effective therapies, thereby preventing further deterioration of performance status before subsequent therapy can be initiated. Our findings may be especially promising in light of the recent approval of the anti-PD-L1 inhibitor avelumab for maintenance treatment after chemotherapy [1]. It can be difficult to determine whether these patients benefit from checkpoint inhibitors using standard imaging as chemotherapy will already have induced a response or stable disease in most patients. Therefore, early response biomarkers would be particularly interesting for this patient group.

## 5. Conclusions

This pilot study indicates that T cell proliferation in peripheral blood during the first weeks of anti-PD-1 is indicative of favorable clinical outcomes in patients with mUC and highlights the value of whole blood transcriptomics for the identification of new biomarkers. Our findings require further validation before implementation in the clinic and will be validated in a prospective, multicenter follow-up study.

## Figures and Tables

**Table 1:** Patient Characteristics.

Total—n (%)	32 (100)
Male—n (%)	25 (78.1)
Age—median (range)	68 (38–80)
ECOG performance score—n (%)	
0	3 (9.4)
1	22 (68.8)
2	7 (21.8)
Treatment—n (%)	
Nivolumab	7 (21.9)
Pembrolizumab	25 (78.1)
Previous platinum-based chemotherapy – n (%)	21 (65.6)
Location of metastases—n (%)	
Lymph node only	9 (28.1)
Visceral metastases	17 (53.1)
Liver metastases	8 (25.0)
Clinical outcome—n (%) *	
PFS < 6 months	13 (40.6)
PFS ≥ 6 months	19 (59.4)
Complete response	5 (15.6)
Partial response	12 (37.5)
Stable disease	1 (3.1)
Not evaluable	1 (3.1)

* according to RECIST1.1

## Data Availability

The data presented in this study are available on request from the corresponding author. The data are not publicly available as they are part of ongoing research into baseline biomarkers for response to checkpoint inhibitors.

## Supplementary Materials

The following are available online at https://www.mdpi.com/article/10.3390/cancers13184660/s1, Figure S1: Gating strategy, Figure S2: Correlations between changes in the identified differentially expressed genes in patients with and without clinical benefit, Figure S3: Changes in DNA replication genes and PDCD1, Figure S4: Cell specificity of cell cycle/DNA replication genes, Figure S5: Changes in absolute lymphocyte counts and the percentage of CD3^+^, CD8^+^ and CD4^+^ T cells, Figure S6. Progression-free survival in patients with and without an above-median increase in Ki-67^+^ CD8^+^ T cells, Figure S7: Correlation between changes in Ki-67^+^ CD8^+^ T cells and changes in the identified differentially expressed genes, Table S1: Antibody details, Supplementary file: identified differentially expressed genes in patients with clinical benefit.
