# Supplementary material for: Whole Blood Transcriptome Profiling Identifies DNA Replication and Cell Cycle Regulation as Early Marker of Response to Anti-PD-1 in Patients with Urothelial Cancer
Source: Cancers (Basel). 2021 Sep 17;13(18):4660. doi: 10.3390/cancers13184660 (PMC8465885; doi:10.3390/cancers13184660)
Supplement: Supplementary file 1 [file cancers-13-04660-s001.zip › supplementary/cancers-1336778-supplementary.pdf]

Supplementary data

Table S1. Antibody details

Antigen	Clone	Conjugate	Company	Catalog No.	Dilution
LAG-3	3DS223H	FITC	eBioscience	#11-2239-42	3/60
TIM-3	F38-2E2	PE	Biolegend	#345006	3/60
Ki-67	Ki-67	PerCP-Cy5.5	Biolegend	#350520	1/10
PD-L1	MIH1	PE-Cy7	BD	#558017	6/60
CTLA-4	BNI3	APC	BD	#555855	6/60
CD4	RPA-T4	APC-R700	BD	#564975	1,5/60
PD-1	EH12.2H7	BV421	BioLegend	#329920	6/60
HLA-DR	G46-6	BV510	BD	#563083	1/60
CD8	G42-8	BV605	BD	#743066	0,8/60
CD3	UCHT1	BV711	Biolegend	#300464	0,8/60

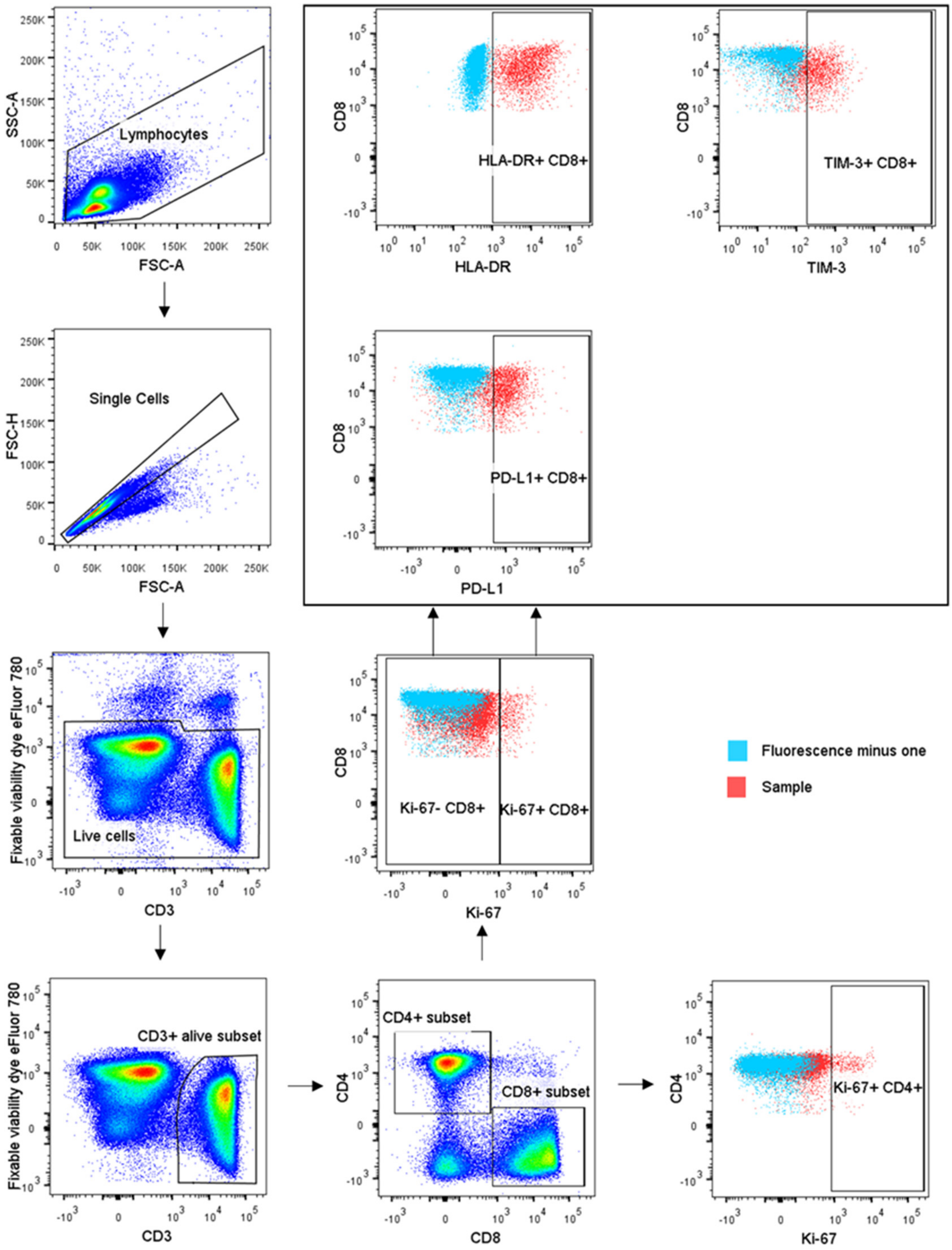

Figure S1. Gating strategy. Gates for Ki-67, HLA-DR, TIM-3 and PD-L1 were set using fluorescence minus one-controls. The CD4^{+} and CD8^{+} cells were both gated to determine the expression of Ki-67 (middle column, third row; only the CD8+ population is shown here).The Ki-67^{+}CD8^{+} and Ki-67^{-}CD8^{+} population were further gated to determine the percentage of cells expression HLA-DR, TIM-3 and PD-L1 (right upper quadrant). In this figure the Ki-67^{+}CD8^{+} population of one representative sample is shown in red. For the Ki-67^{-}CD8^{+} population (not shown) the same gates were used.

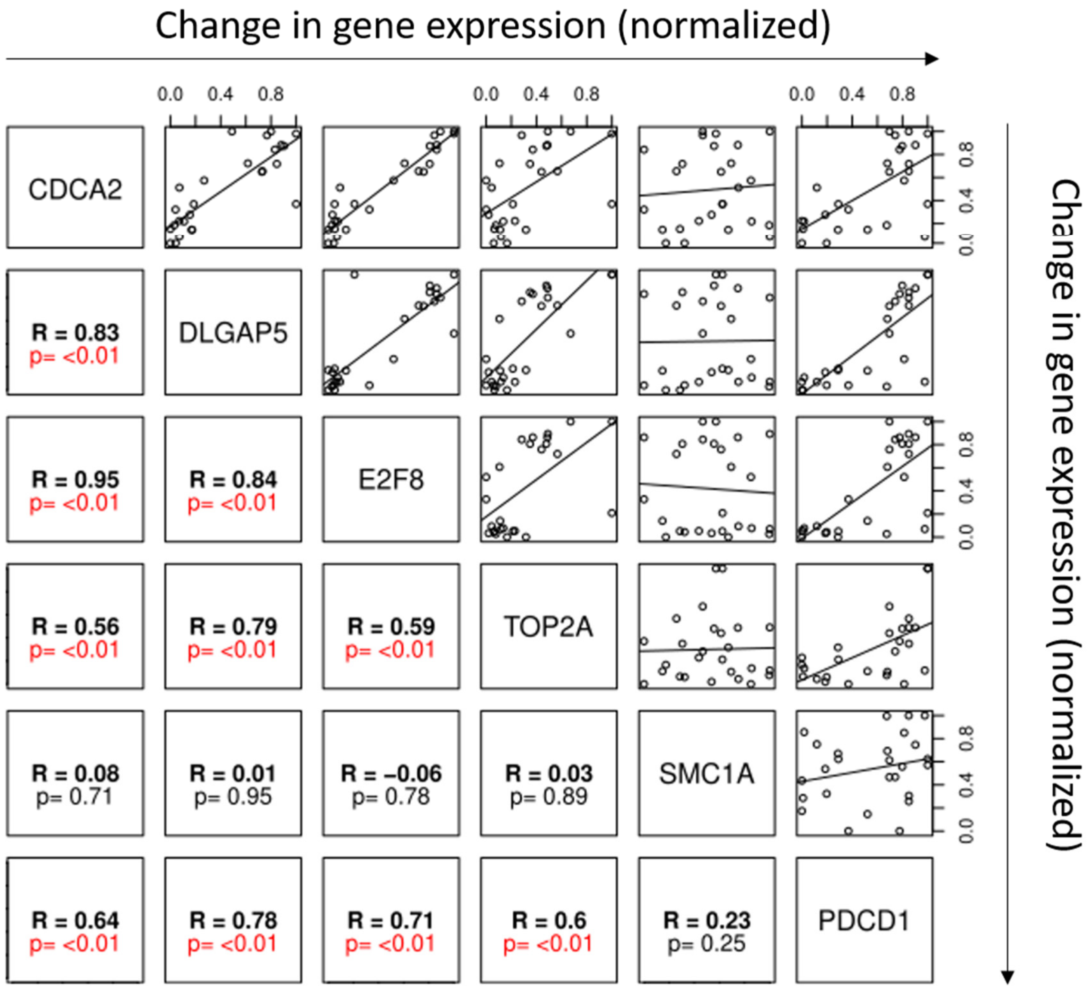

Figure S2. Correlations between changes in the identified differentially expressed genes in patients with and without clinical benefit. The matrix depicts correlations between pairs of variables shown in the matrix diagonal, with the scatter plots in the upper triangular and the statistics in the lower triangular referring to the same pair of variables. Data were normalized using the following formula: (x – min(x))/(max(x)-min(x)). Correlations were analyzed using the Pearson's correlation coefficient. Significant correlations are highlighted in red.

.

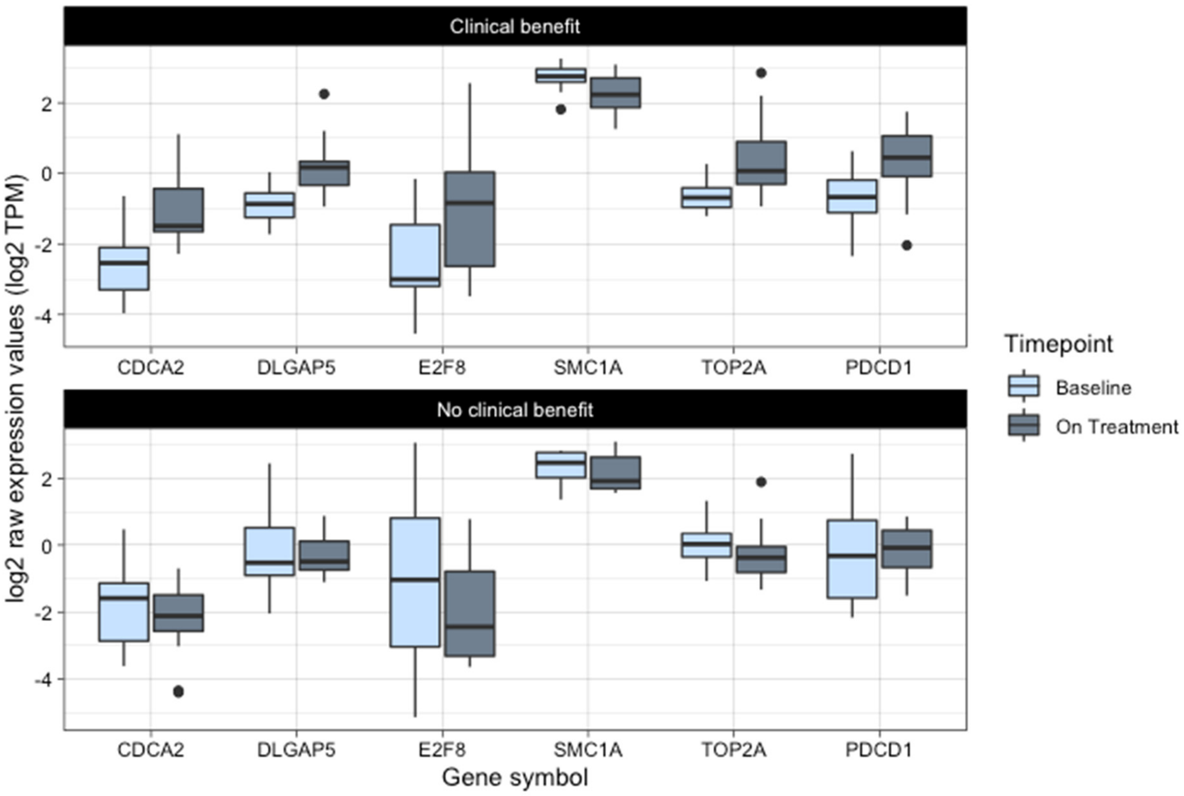

Figure S3 Changes in DNA replication genes and PDCD1. The upper panel depicts changes in patients with clinical benefit after 1-2 cycles of anti-PD-1. Below, changes in patients without clinical benefit are shown.

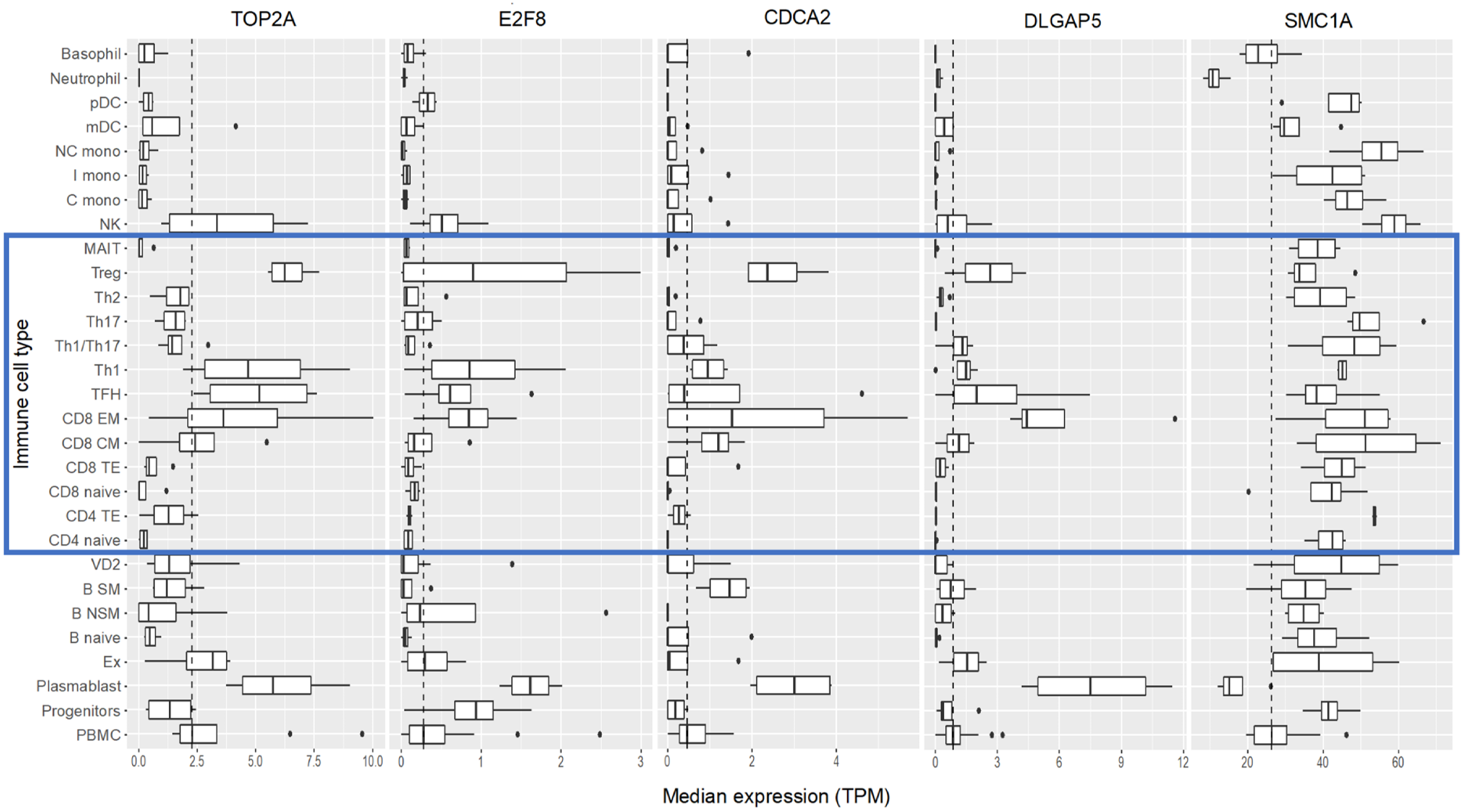
TOP2A
E2F8
CDCA2
DLGAP5
SMC1A
Immune cell type
Basophil
Neutrophil
pDC
mDC
NC mono
I mono
C mono
NK
MAIT
Treg
Th2
Th17
Th1/Th17
Th1
TFH
CD8 EM
CD8 CM
CD8 TE
CD8 naive
CD4 TE
CD4 naive
VD2
B SM
B NSM
B naive
Ex
Plasmablast
Progenitors
PBMC
Median expression (TPM)

Figure S4. Cell specificity of cell cycle/DNA replication genes. A publicly available dataset (GSE107011[27]) was used to evaluate in which immune cell subsets the differentially expressed DNA replication/cell cycle genes (DEGs) are expressed. The dotted line indicates median expression in unsorted peripheral blood mononuclear cells. All T cell subsets are delineated by a blue rectangle. The expression of the four upregulated DEGs was enhanced in several T cell subsets, particularly in the regulatory T cells (Tregs), T-helper 1 (Th1) cells, follicular helper T cells (TFH) and $CD8^+$ effector memory (CD8 EM) cells. SMC1A was highly expressed in nearly all immune cell subsets, with no particular specificity.

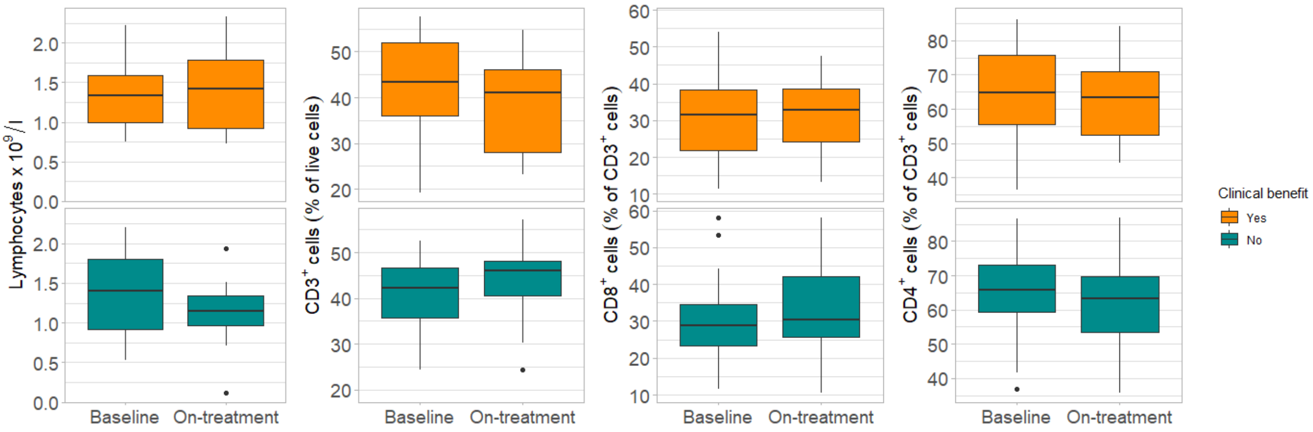

Figure S5 Changes in absolute lymphocyte counts and the percentage of $CD3^+$, $CD8^+$ and $CD4^+$ T cells.

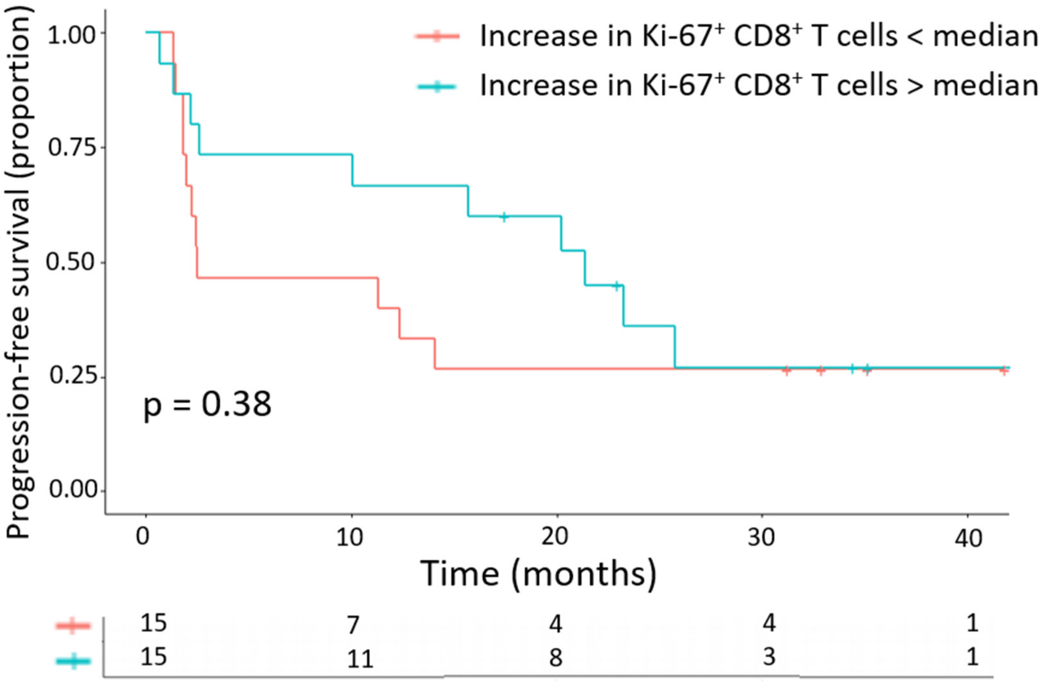

Figure S6. Progression-free survival in patients with and without an above-median increase in Ki-67^{+} CD8+ T cells

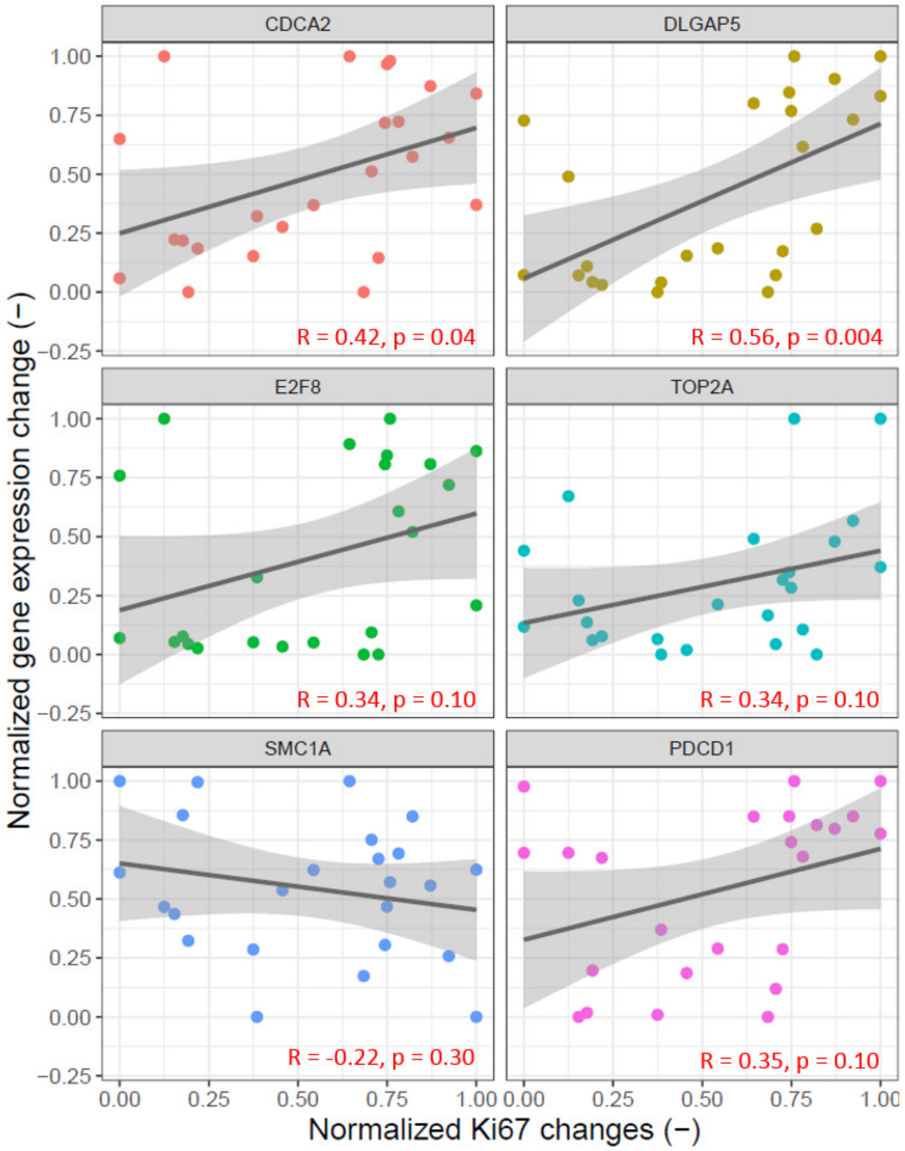

Figure S7. Correlation between changes in Ki-67$^+$ CD8$^+$ T cells and changes in the identified differentially expressed genes. Data were normalized using the following formula: x – min(x)/(max(x)-min(x). Correlation coefficients and p-values are highlighted in red.
